# Immune responses to stress after stress management training in patients with rheumatoid arthritis

**DOI:** 10.1186/ar4390

**Published:** 2013-11-26

**Authors:** Sabine JM de Brouwer, Henriët van Middendorp, Floris W Kraaimaat, Timothy RDJ Radstake, Irma Joosten, A Rogier T Donders, Agnes Eijsbouts, Saskia Spillekom-van Koulil, Piet LCM van Riel, Andrea WM Evers

**Affiliations:** 1 Department of Medical Psychology, Radboud University Medical Center, P.O. Box 9101, 6500 HB Nijmegen, the Netherlands; 2 Department of Rheumatology, Radboud University Medical Center, P.O. Box 9101, 6500 HB Nijmegen, the Netherlands; 3 Department of Rheumatology and Clinical Immunology & Laboratory of Translational Immunology, University Medical Center Utrecht, P.O. Box 85500, 3508 GA Utrecht, the Netherlands; 4 Department of Laboratory Medicine, Radboud University Medical Center, P.O. Box 9101, 6500 HB Nijmegen, the Netherlands; 5 Department for Health Evidence, Radboud University Medical Center, P.O. Box 9101, 6500 HB Nijmegen, the Netherlands; 6 Department of Rheumatology, Sint Maartenskliniek, P.O. Box 9011, 6500 GM Nijmegen, the Netherlands; 7 Department of Clinical, Health, and Neuropsychology, Leiden University, P.O. Box 9555, 2300 RB Leiden, the Netherlands

## Abstract

**Introduction:**

Psychological stress may alter immune function by activating physiological stress pathways. Building on our previous study, in which we report that stress management training led to an altered self-reported and cortisol response to psychological stress in patients with rheumatoid arthritis (RA), we explored the effects of this stress management intervention on the immune response to a psychological stress task in patients with RA.

**Methods:**

In this study, 74 patients with RA, who were randomly assigned to either a control group or a group that received short stress management training, performed the Trier Social Stress Test (TSST) 1 week after the intervention and at a 9-week follow-up. Stress-induced changes in levels of key cytokines involved in stress and inflammatory processes (for example, interleukin (IL)-6 and IL-8) were assessed.

**Results:**

Basal and stress-induced cytokine levels were not significantly different in patients in the intervention and control groups one week after treatment, but stress-induced IL-8 levels were lower in patients in the intervention group than in the control group at the follow-up assessment.

**Conclusions:**

In line with our previous findings of lower stress-induced cortisol levels at the follow-up of stress management intervention, this is the first study to show that relatively short stress management training might also alter stress-induced IL-8 levels in patients with RA. These results might help to determine the role of immunological mediators in stress and disease.

**Trial registration:**

The Netherlands National Trial Register (NTR1193)

## Introduction

Psychological stress may alter immune function by activating physiological pathways of stress, such as the autonomic nervous system and the hypothalamus–pituitary–adrenal axis, which in turn interact with the immune system [1-4]. Consequently, stress could have negative effects on health, particularly in populations with immune dysfunction, such as patients with rheumatoid arthritis (RA). The pathophysiological mechanisms involved in stress and disease exacerbation have not yet been elucidated.

Psychological responses to stress that might lead to immune dysregulation can be altered by interventions aimed at reducing psychological stress [1,5]. As yet there is no consensus about whether and to what extent stress management interventions are able to alter immune function. In an extensive meta-analysis by Miller and Cohen there was only modest evidence that different types of stress management interventions change basal immune function in healthy and clinical populations, with most consistent changes being found in basal total leukocyte counts and secretory immunoglobulin A levels [6]. More recent studies reported that psychological interventions for patients with HIV or cancer changed basal lymphocyte proliferation and basal levels of proinflammatory cytokines [7-10]. Even though the effects of psychological interventions in patients with RA have been extensively studied and reviewed [11-16], there are only incidental reports of immune changes after psychological interventions in patients with RA, such as changes in interleukin (IL)-6 or interferon-gamma (IFNγ) [17,18], or in immune measures indicative of disease status, such as C-reactive protein and erythrocyte sedimentation rate [19-24]. Potentially, previous effects in RA might be limited because changes in immune function in response to a real-life stressor have not yet been investigated combining both a stress management intervention and a stress induction paradigm. Particularly then, the benefits of stress management training can become evident because patients are challenged to cope with a stressful situation.

We previously showed that a short course of stress management training decreased the subjective distress response and stress-induced cortisol levels in patients with RA at a follow-up assessment, and especially in those patients psychologically at risk [5]. In the present study, we explored the effects of the intervention on stress-induced levels of key cytokines involved in disease progression (for example, IL-6 and IL-8) in patients with RA, with stress being elicited by the Trier Social Stress Test. Building on our previous findings [5], we expected that patients in the intervention group would show an altered cytokine response to acute psychosocial stress compared with controls at the 9-week follow-up assessment. We also explored immune effects specifically in patients psychologically at risk.

## Materials and methods

This study was part of a larger trial for which the methods and CONSORT statement have been described extensively elsewhere [5]. The study protocol was approved by the regional medical ethics committee (CMO Region Arnhem-Nijmegen) and was registered in The Netherlands National Trial Register (NTR 1193). Written informed consent was obtained from all participants.

### Participants and procedure

#### Participants

Ninety-six eligible patients with RA [25] were randomized to one of two parallel groups: the control or the intervention condition. After randomization, 19 participants withdrew before the first stress test and three participants were excluded based on our predefined exclusion criteria (that is, use of psychiatric medication). In addition, seven out of 74 participants withdrew before the second stress test. Reasons for withdrawal were physical comorbidity, severe illness or death of a significant other, a change in pharmacotherapy, or lack of motivation (for more information on completers and dropouts, see the flowchart in [5]). There were no differences in sociodemographic variables and psychological and physical functioning at baseline between the dropouts and the completers. For explorative subgroup analyses of patients psychologically at risk, participants were *post hoc* divided into two subgroups using a median split of a composite score for baseline anxiety and negative mood [5].

#### Study design

Participants performed a stress test 3 weeks after the first assessment (post treatment) and 9 weeks thereafter (follow-up). One-half of the participants had participated in an individual stress management training program between the first and second assessments. The control group received care as usual. Stress test sessions were run between 13:00 and 15:30 hours. Participants refrained from using caffeine, alcohol, nicotine, or physical exercise on the test day, and from eating 2 hours before the first blood sample was drawn. Forty minutes before the stress test, a venous catheter was inserted into the nondominant arm and participants rested for 20 minutes. Blood samples were taken at baseline (that is, after 20 minutes of rest), immediately after the stress test, and 20 and 60 minutes later (*t* = 0, *t* = 20, *t* = 40, and *t* = 80 minutes, respectively).

#### Stress task

The Trier Social Stress Test is a standardized laboratory stress task consisting of a mock job interview and mental arithmetic, and induces self-reported, neuroendocrine, and autonomic nervous system responses [26,27].

#### Stress management training

Participants in the intervention group received individual stress management training as described previously [5]. The program consisted of four individual 1-hour sessions of stress management with a trained therapist over 2 consecutive weeks and included applied, progressive, cue-controlled, and differential relaxation techniques, as well as psycho-education, breathing and visualization exercises. After the training, patients were encouraged to stick to a relapse-prevention checklist during the 9-week follow-up period.

### Measures

This study builds on a previous study [5], in which general psychological (for example, anxiety), physical (28-joint Disease Activity Score), autonomic (alpha-amylase) and neuroendocrine (cortisol) outcomes are reported, by further exploring immune responses to stress through measurement of various circulating cytokines.

#### Cytokine assay

The blood samples that were collected during the two stress tests (post treatment and follow-up) were stored at –35°C until analysis. Based on the literature of psychophysiological stress reactivity in healthy populations and chronic inflammatory diseases, such as RA [1,28-30], human IL-1β, IL-2, IL-4, IL-5, IL-6, IL-7, IL-8, IL-10, IFNγ and tumor necrosis factor alpha (TNFα) were measured in serum using human cytokine multiple kits (Invitrogen Corporation, Camarillo, CA, USA) according to the manufacturer’s instructions. Samples were analyzed with a Luminex® 100 TM instrument (Luminex Corporation, Austin, TX, USA). The sensitivity of the cytokine assay was <5 pg/ml for all cytokines measured. To reduce error variance caused by between-run variation, all samples from one participant were analyzed in the same run.

### Statistical analysis

Data for the 74 participants who completed the study protocol were analyzed. Skewed data were logarithmically transformed to generate unskewed data distributions before statistical analysis. Normal distributions and residuals were not obtained after logarithmic transformation of data for IL-5 and IFNγ levels. Between-group differences in age, sex, education, and psychological measures at baseline were tested with independent Student’s *t* tests and chi-square analyses. Baseline cytokine levels (*t* = 0 minutes) were compared between intervention and control groups with analyses of covariance. Cytokine responses to the Trier Social Stress Test (post treatment and follow-up) were evaluated using a linear mixed model taking into account the specific design features of the study. Cytokine levels (IL-1β, IL-2, IL-4, IL-5, IL-6, IL-7, IL-8, IL-10, IFNγ, and TNFα) were used as dependent variables; and group, baseline measurement of the dependent variable (*t* = 0 minutes), and time (*t* = 20 minutes, *t* = 40, minutes, and *t* = 80 minutes) were used as independent variables. As group by time interactions were not observed, the final model contained only main effects. Explorative subgroup analyses were performed to test whether effects were particularly detected in patients psychologically at risk as compared with patients not at risk [5] by incorporating risk group and risk group by treatment interactions into the models. A significant interaction was interpreted as indicating that there were subgroup differences in the effect of the treatment. Stratified analyses were performed to gain a better understanding of the nature of the responses in the patient subgroups. For each outcome measure, an unstructured covariance matrix was used to model the dependence between repeated measurements of the dependent variable. Owing to (a tendency towards) an unequal sex distribution, use of hormonal contraceptives, baseline anxiety scores, and the use of nonsteroidal anti-inflammatory drugs across the two groups [5], all analyses were performed with these four covariates.

Because participants dropped out mostly prior to the first stress test (see previous section and [5]), and consequently no stress test data were available for these participants 1 week after treatment and at follow-up, intention-to-treat analyses were not performed [31]. In total, the data for the 74 patients (post treatment) and 67 patients (follow-up) included in the analyses were 85% complete, mainly because a venous catheter could not be inserted in a number of patients during one or both stress tests. Cytokines were significantly intercorrelated with at least five to nine of the other cytokines, and significant correlations ranged from 0.20 to 0.80. Undetectable levels (in percentage of available samples) of IL-1β (33%), IL-2 (41%), IL-4 (37%), IL-5 (43%), IL-6 (27%), IL-7 (41%), IL-8 (13%), IL-10 (25%), IFNγ (70%) and TNFα (16%) were set to zero and included in all analyses. The Bonferroni correction for multiple testing was not applied due to the explorative nature of this study, the small sample size, and the high intercorrelation of most cytokines, which makes the method even more conservative than in other applications [32]. Analyses were performed using SPSS 16.0 for Windows (SPSS Inc, Chicago, IL, USA). For all analyses, the significance level was *α* = 0.05 (two-sided).

## Results

### Psychophysiological stress reactivity

#### Cytokine levels at baseline

Both after the intervention and at follow-up there were no significant differences between the intervention and control groups in baseline levels (*t* = 0 minutes) of all cytokines (*P* >0.05) (Table 1 and Additional file 1).

**Table 1:** Baseline and stress-induced cytokine levels (pg/ml) in the intervention and control conditions post treatment and at follow-up

		***t *** **= 0 minutes**	***t *** **= 20 minutes**	***t *** **= 40 minutes**	***t *** **= 80 minutes**
**IL-1β**					
Post-treatment	IC	92.39 (33.6)	106.7 (41.4)	111.3 (46.4)	111.6 (45.4)
	CC	114.3 (38.6)	130.9 (43.5)	116.6 (37.0)	136.7 (59.0)
Follow-up	IC	66.48 (18.8)	77.43 (23.7)	69.49 (20.1)	70.89 (19.9)
	CC	119.0 (43.0)	100.7 (32.5)	98.13 (34.1)	117.6 (41.2)
**IL-2**					
Post-treatment	IC	28.11 (11.1)	30.85 (11.9)	34.83 (15.6)	31.58 (13.4)
	CC	32.60 (14.0)	42.86 (19.2)	38.77 (17.3)	47.51 (27.0)
Follow-up	IC	20.28 (7.20)	25.27 (10.3)	17.37 (7.23)	19.32 (7.15)
	CC	35.89 (18.4)	27.82 (14.1)	28.12 (14.0)	32.24 (15.4)
**IL-4**					
Post-treatment	IC	48.22 (24.9)	48.30 (21.0)	48.98 (20.3)	52.90 (25.7)
	CC	60.18 (29.3)	72.79 (30.1)	65.76 (28.4)	65.39 (29.9)
Follow-up	IC	33.22 (13.9)	35.16 (14.9)	33.62 (12.6)	33.08 (13.6)
	CC	56.48 (23.2)	51.79 (20.0)	50.91 (21.5)	56.99 (24.8)
**IL-5**					
Post-treatment	IC	20.94 (15.2)	21.89 (15.3)	23.24 (16.5)	21.49 (15.6)
	CC	5.129 (2.17)	7.887 (3.72)	7.006 (2.94)	8.948 (4.51)
Follow-up	IC	16.23 (12.8)	15.89 (12.1)	15.14 (12.1)	17.60 (14.5)
	CC	7.368 (3.48)	6.048 (2.95)	5.929 (2.60)	6.969 (3.11)
**IL-6**					
Post-treatment	IC	36.46 (11.2)	34.94 (9.69)	38.80 (11.1)	40.36 (11.2)
	CC	30.18 (9.36)	41.18 (15.2)	33.54 (10.7)	39.57 (16.2)
Follow-up	IC	25.79 (7.51)	23.39 (7.90)	23.63 (6.41)	23.18 (6.68)
	CC	31.20 (14.1)	30.18 (12.0)	29.22 (11.3)	30.43 (11.6)
**IL-7**					
Post-treatment	IC	70.11 (26.0)	70.27 (24.8)	73.62 (24.7)	69.51 (27.2)
	CC	52.69 (16.5)	63.65 (20.5)	55.94 (18.5)	60.73 (21.8)
Follow-up	IC	52.54 (23.4)	54.54 (23.5)	49.13 (19.6)	53.32 (22.2)
	CC	52.80 (18.4)	53.69 (18.0)	48.64 (18.2)	52.89 (19.1)
**IL-8**					
Post-treatment	IC	22.14 (3.67)	16.19 (3.70)	17.02 (3.53)	14.47 (3.41)
	CC	29.55 (6.50)	34.04 (7.81)	29.45 (6.75)	28.18 (7.14)
Follow-up	IC	19.51 (4.28)	13.97 (4.67)	12.53 (4.00)	10.37 (3.38)^a^
	CC	33.63 (6.68)	33.46 (8.33)	29.31 (7.83)	23.64 (5.72)
**IL-10**					
Post-treatment	IC	172.0 (75.2)	183.7 (81.0)	175.5 (80.0)	164.7 (75.8)
	CC	49.33 (25.9)	57.07 (31.8)	73.55 (48.1)	71.02 (45.9)
Follow-up	IC	72.67 (35.6)	115.6 (54.6)	85.90 (41.9)	78.49 (37.4)
	CC	46.83 (26.8)	43.63 (24.2)	39.90 (20.9)	50.27 (28.0)
**IFNγ**					
Post-treatment	IC	1.063 (0.39)	1.070 (0.43)	0.821 (0.33)	0.706 (0.31)
	CC	1.545 (0.45)	8.328 (4.98)	6.623 (4.91)	7.591 (5.70)
Follow-up	IC	0.857 (0.39)	0.772 (0.32)	0.925 (0.36)	1.062 (0.38)
	CC	4.389 (2.87)	8.535 (6.37)	6.597 (4.98)	7.377 (5,94)
**TNFα**					
Post-treatment	IC	31.40 (8.54)	34.98 (8.95)	34.82 (8.35)	35.60 (10.7)
	CC	34.56 (12.4)	39.35 (12.7)	36.00 (11.1)	35.45 (11.2)
Follow-up	IC	23.90 (5.75)	26.44 (6.94)	24.74 (5.89)	25.19 (6.44)
	CC	26.26 (8.74)	23.84 (7.17)	25.04 (9.44)	25.93 (8.92)

Data presented as mean (± standard error of the mean). CC, control condition; IC, intervention condition; IFNγ, interferon gamma; IL, interleukin; TNFα, tumor necrosis factor alpha. ^a^Significant between-group effect (*P* ≤0.05). Means at the four time points. Statistical analyses performed on ln-transformed data.

#### Post-treatment stress-induced cytokine levels

Immediately after the intervention, stress-induced cytokine levels were similar in the intervention and control groups (group effect, *P* >0.10 for all cytokines), indicating that patients in the intervention group did not have an altered immune response to stress compared with patients in the control group. Subgroup analyses also showed no interaction between condition (intervention/control) and psychological risk group (high/low) (*P* >0.20 for all cytokines) (Table 1 and Additional file 1).

#### Follow-up stress-induced cytokine levels

**Figure 1:**
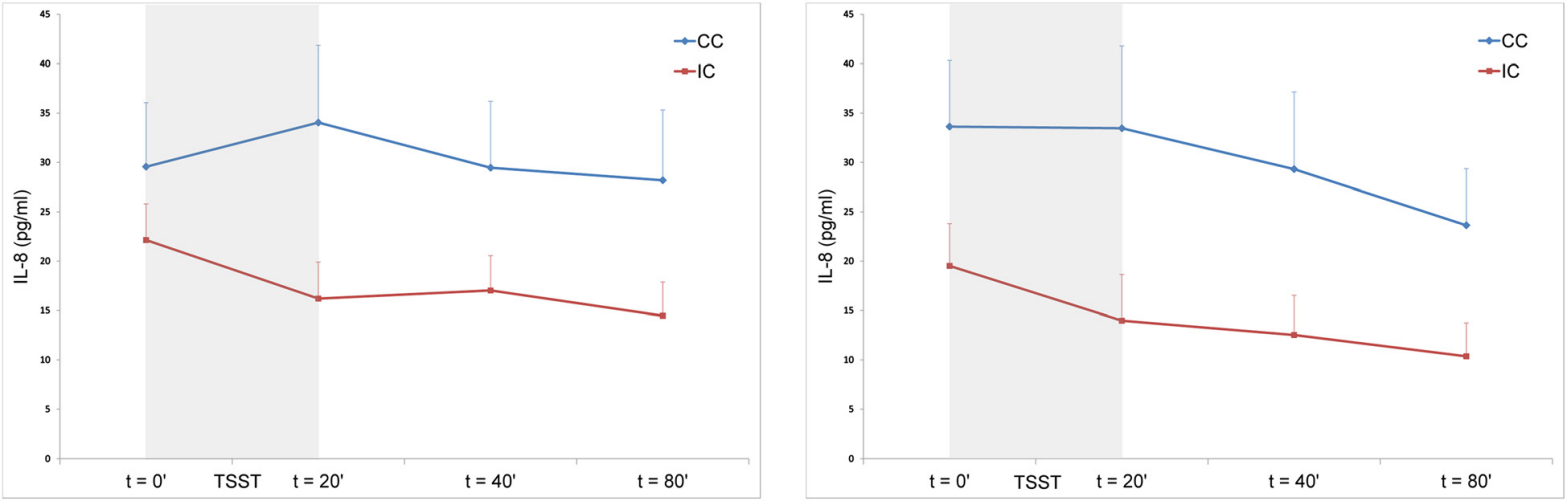
**Interleukin-8 response to stress.** Mean ± standard error of the mean interleukin (IL)-8 levels (pg/ml) at *t* = 0 minutes (baseline/pre Trier Social Stress Test (TSST)), *t* = 20 minutes, *t* = 40 minutes, and *t* = 80 minutes (post TSST) of patients in the intervention conditions (IC) and control conditions (CC) immediately after the intervention (left: IC, *n* = 35; CC, *n* = 32) and at follow-up (right: IC, *n* = 33; CC, *n* = 28).

At the follow-up assessment, stress-induced IL-8 levels were significantly lower in patients in the intervention group than in patients in the control group (group effect, *F*(1, 54.273) = 5.421, *P* = 0.02) (Figure 1). Exploration of IL-8 responses in subgroups of patients psychologically at risk and not at risk showed a tendency towards an interaction effect between condition (intervention/control) and risk group (high/low) (interaction effect, *F*(1, 51.990) = 3.244, *P* = 0.08), indicating that high-risk patients tended to respond differently to stress management training than low-risk patients. *Post hoc* tests revealed that IL-8 levels were more decreased in high-risk patients in the intervention group than in the low-risk intervention group. Omission of the data for patients with undetectable IL-8 levels from analyses did not change the main result (group effect, *F*(1, 48.178) = 8.226, *P* = 0.01), and high-risk patients still showed a (significantly) different response to the training than low-risk patients (interaction effect condition and risk status, *F*(1, 46.212) = 4.472, *P* = 0.04). For all other cytokines, there were no significant differences in levels after stress induction between the intervention and control groups at follow-up (*P* >0.10) (Table 1 and Additional file 1).

## Discussion

This is the first study to explore the response of circulating cytokines to a psychosocial stress test after stress management training in patients with RA. Although no differences in basal and stress-induced levels of key cytokines were observed immediately after the intervention, patients in the intervention group had lower stress-induced IL-8 levels than patients in the control group at the follow-up assessment. Results suggest that a short individual training in stress management might alter immune parameters after a psychosocial stress task in a population with immune dysfunction; namely, patients with RA. This finding is in line with our previous report indicating that the stress management training improves psychological functioning and influences subjective and endocrine parameters of stress (that is, distress and cortisol levels) at the follow-up assessment [5].

Stress-induced immune effects after a stress management intervention have not so far been investigated in rheumatic patients, including patients with RA. Stress induction paradigms using only a single stress exposure have yielded relatively robust effects on IL-6, IL-1β, and IFNγ levels in various healthy and patient populations [28,29]. Stress exposure also changes levels of these and other cytokines in rheumatic patients, but results are much less consistent [2]. For example, IL-6 levels increased in response to a cold pressor task in patients with RA and juvenile idiopathic arthritis [33,34], but IL-6 and IFNγ levels remained unchanged after psychological stress was induced in patients with RA and systemic lupus erythematosus [35-37]. Differences in stress induction paradigms and detection methods used and differences in the heterogeneity of patient samples might explain the inconsistent findings. Immune function after stress management training has only been measured incidentally in patients with RA and, moreover, has not been investigated in combination with stress exposure. One study reported altered basal IFNγ levels after emotional disclosure therapy for patients with RA [17], while lower basal IL-6 levels were observed after cognitive behavioral therapy compared with meditation and education groups [18]. Several other studies also reported other types of biological markers, mostly erythrocyte sedimentation rate and/or C-reactive protein, often as part of assessing overall disease activity, but did not find intervention-related changes [21,22,24,38-46]. In our study, the stress management intervention did not change basal or stress-induced cytokine levels, except for a decrease in stress-induced IL-8 levels at follow-up.

Chemotactic IL-8 is a key player in the acute exacerbation of inflammatory conditions, directing neutrophils and other cell types (for example, monocytes and lymphocytes) to sites of inflammation when homeostasis is disrupted [47]. Blocking the actions of IL-8 has been shown to prevent acute inflammation in animal models [48]. The lipopolysaccharide-stimulated production of IL-8 has been found to be positively correlated with perceived stress in healthy adults, and this could be primarily attributed to negative affect [49,50]. However, IL-8 levels did not change after the induction of stress with the cold pressor task in patients with juvenile idiopathic arthritis and healthy controls [34]. Whether IL-8 acts as a more general marker of stress or whether it is specifically involved in the physiological stress response of patients with RA is not yet clear. Consequently, future studies should compare IL-8 responses to stress and stress management training in both healthy and clinical populations. Interestingly, the effect of the stress management training on stress-induced IL-8 levels tended to be particularly evident in patients with heightened levels of anxiety and negative mood. We found comparable effects for self-reported levels of tension and cortisol levels in our previous report [5], but these measures were not related to IL-8 levels in this study. In addition, the effectiveness of psychological treatment for RA patients at risk was reported previously [51], which warrants further research into the benefits of stress management on different types of psychophysiological parameters in high-risk patients.

This study had several limitations. The relatively homogeneous and small sample of patients with mild RA prevents generalization of our findings. The normal range for many immune parameters is very broad and psychological interventions, especially of short duration, might not induce physiological changes of sufficient magnitude or duration to move cytokine levels beyond this range [6]. Nevertheless, intervention studies have demonstrated that immune alterations occur when people display a change in cognition [52] and emotion [7]. Moreover, intervention-related immune changes could have been masked by biological forces, such as disease flare-ups and biological treatments that affect the patients’ immune system [6]. Although we tried to limit effects of disease flare-ups by monitoring the patients’ disease status and ruled out that treatment effects were caused by differences in biological treatment protocols through covariate analyses, we cannot preclude that this problem might have influenced our results. Prompted by earlier unequivocal findings of stress-induced changes to immune function in rheumatic patients [2], the high intercorrelation of most cytokines, and the small sample size, Bonferroni correction for multiple testing was not applied in this explorative study. Future research should try to replicate our findings and, if possible, apply the Bonferroni correction to data with large sample sizes. Moreover, the direction of other cytokine responses observed in this study (for example, IFNγ) seems consistent with the stress literature and tentatively suggests a broader effect of stress management training on immune function, but larger studies are needed to validate this effect. Furthermore, no statements can be made about the clinical relevance of our results, especially since the intervention was of short duration (four 1-hour sessions over 2 weeks) and disease activity did not improve over the course of our study [5]. A longer intervention that may produce more pronounced effects might overcome these problems. Another general problem concerning immune markers in stress research, particularly circulating cytokines, is the ambiguity regarding the interpretation of findings. Circulating levels of cytokines are thought to reflect levels of systemic inflammation and are correlated with disease activity and radiographic progression [53]; however, changes in cytokine concentrations from baseline might not indicate *de novo* cytokine production or clearance, but a redistribution of existing cytokines from or into the periphery [54]. To what extent these alterations represent adaptive or maladaptive immune processes is not well understood and needs further investigation.

## Conclusions

Patients with RA who received training in stress management not only show changes in the subjective and cortisol response to stress [5], but might also be characterized by an altered immune response to stress; that is, lower IL-8 levels. Although results of this and our previous study need validation in larger studies, they provide preliminary evidence that a short psychological intervention is not only able to improve psychological functioning, but also acts on the neuroendocrine and immune systems and therefore might have the potential to ameliorate the possible harmful effects of stress on health in patients with RA. Stress management training might prove to be beneficial as an adjunct to standard therapy to control arthritis symptoms.

## Abbreviations

IFNγ: Interferon gamma; IL: Interleukin; RA: Rheumatoid arthritis; TNFα: Tumor necrosis factor alpha.

## Competing interests

The authors declare that they have no competing interests.

## Authors’ contributions

SJMdB, AWME, PLCMvR and FWK conceived and designed the experiments. SJMdB, AWME, SS-vK and AE were involved in the acquisition of data. SJMdB, AWME, ARTD, TRDJR, IJ and HvM contributed to the analyses and interpretation of data. SJMdB and AWME drafted the manuscript. All authors critically reviewed and approved the final manuscript.

## Supplementary Material

Additional file 1 **is Figure S1 showing the mean response to stress of (A) IL-1β, (B) IL-2, (C) IL-4, (D) IL-5, (E) IL-6, (F) IL-7, (G) IL-8, (H) IL-10, (I) IFNγ, and (J) TNFα (in pg/ml ± standard error of the mean) at *t *** **= 0 minutes (baseline/pre TSST), *t *** **= 20 minutes, *t *** **= 40 minutes, and *t *** **= 80 minutes (post TSST) for patients in the intervention condition (IC) and control condition (CC) immediately after the intervention (post; red) and at follow-up (FU; blue).** Click here for file
