# Supplementary figures and images for: Immune responses to stress after stress management training in patients with rheumatoid arthritis
Source: Arthritis Res Ther. 2013 Nov 26;15(6):R200. doi: 10.1186/ar4390 (PMC3978719; doi:10.1186/ar4390)

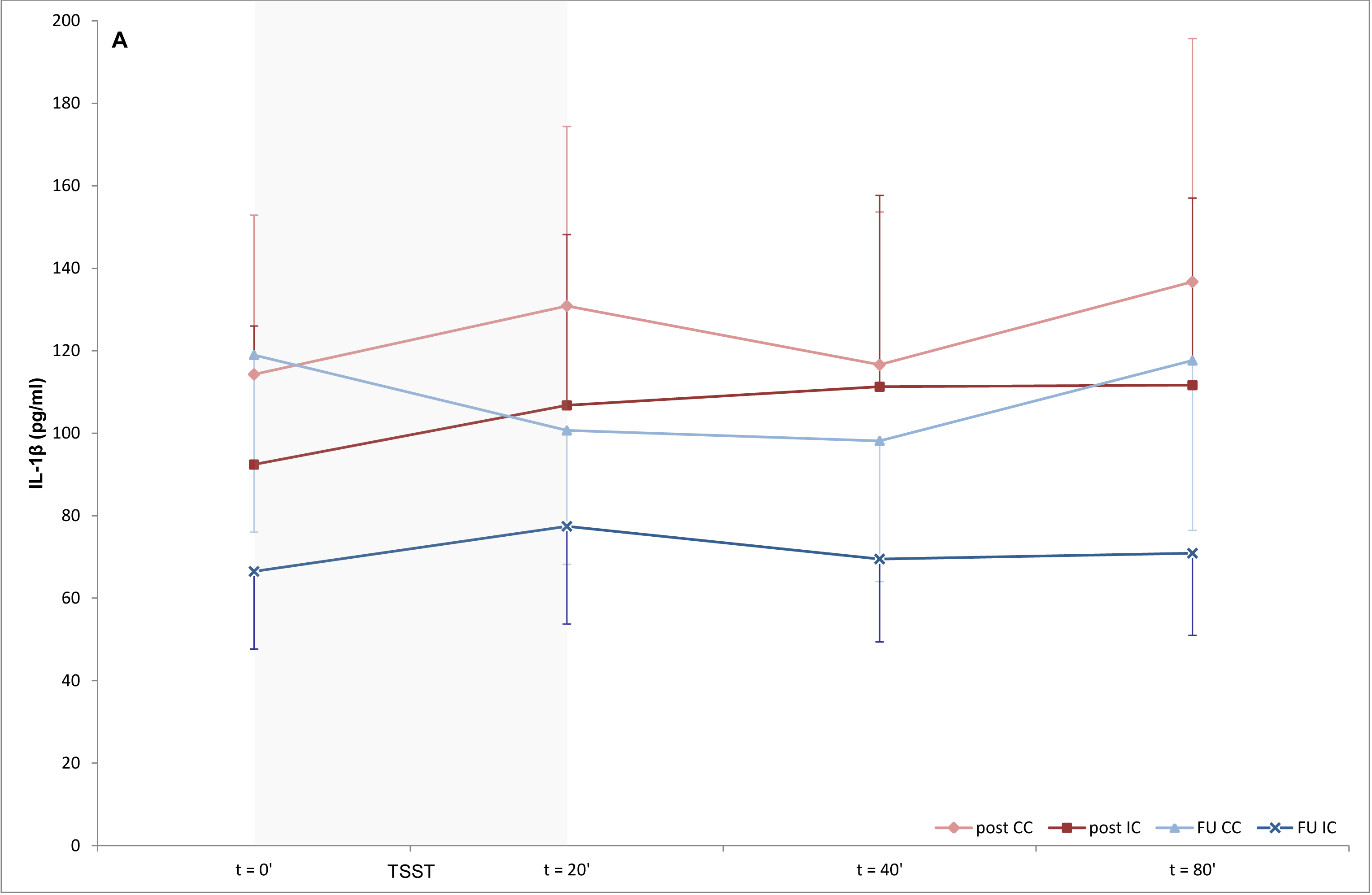

Supplement: Additional file 1 — is Figure S1 showing the mean response to stress of (A) IL-1β, (B) IL-2, (C) IL-4, (D) IL-5, (E) IL-6, (F) IL-7, (G) IL-8, (H) IL-10, (I) IFNγ, and (J) TNFα (in pg/ml ± standard error of the mean) at t = 0 minutes (baseline/pre TSST), t = 20 minutes, t = 40 minutes, and t = 80 minutes (post TSST) for patients in the intervention condition (IC) and control condition (CC) immediately after the intervention (post; red) and at follow-up (FU; blue). [file ar4390-S1.zip › Additional file 1/4354025511000446_add1.tiff]

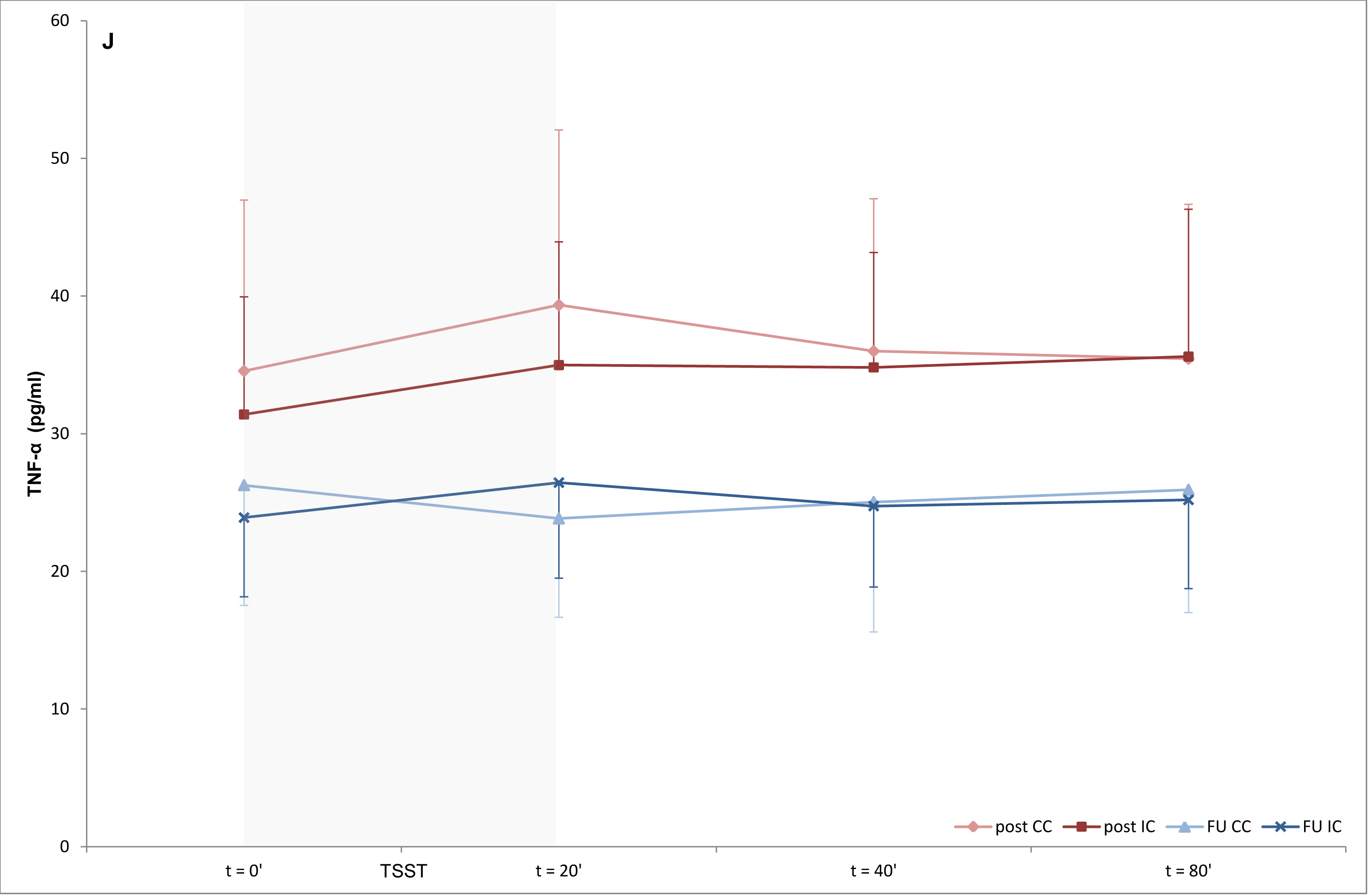

Supplement: Additional file 1 — is Figure S1 showing the mean response to stress of (A) IL-1β, (B) IL-2, (C) IL-4, (D) IL-5, (E) IL-6, (F) IL-7, (G) IL-8, (H) IL-10, (I) IFNγ, and (J) TNFα (in pg/ml ± standard error of the mean) at t = 0 minutes (baseline/pre TSST), t = 20 minutes, t = 40 minutes, and t = 80 minutes (post TSST) for patients in the intervention condition (IC) and control condition (CC) immediately after the intervention (post; red) and at follow-up (FU; blue). [file ar4390-S1.zip › Additional file 1/4354025511000446_add10.tiff]

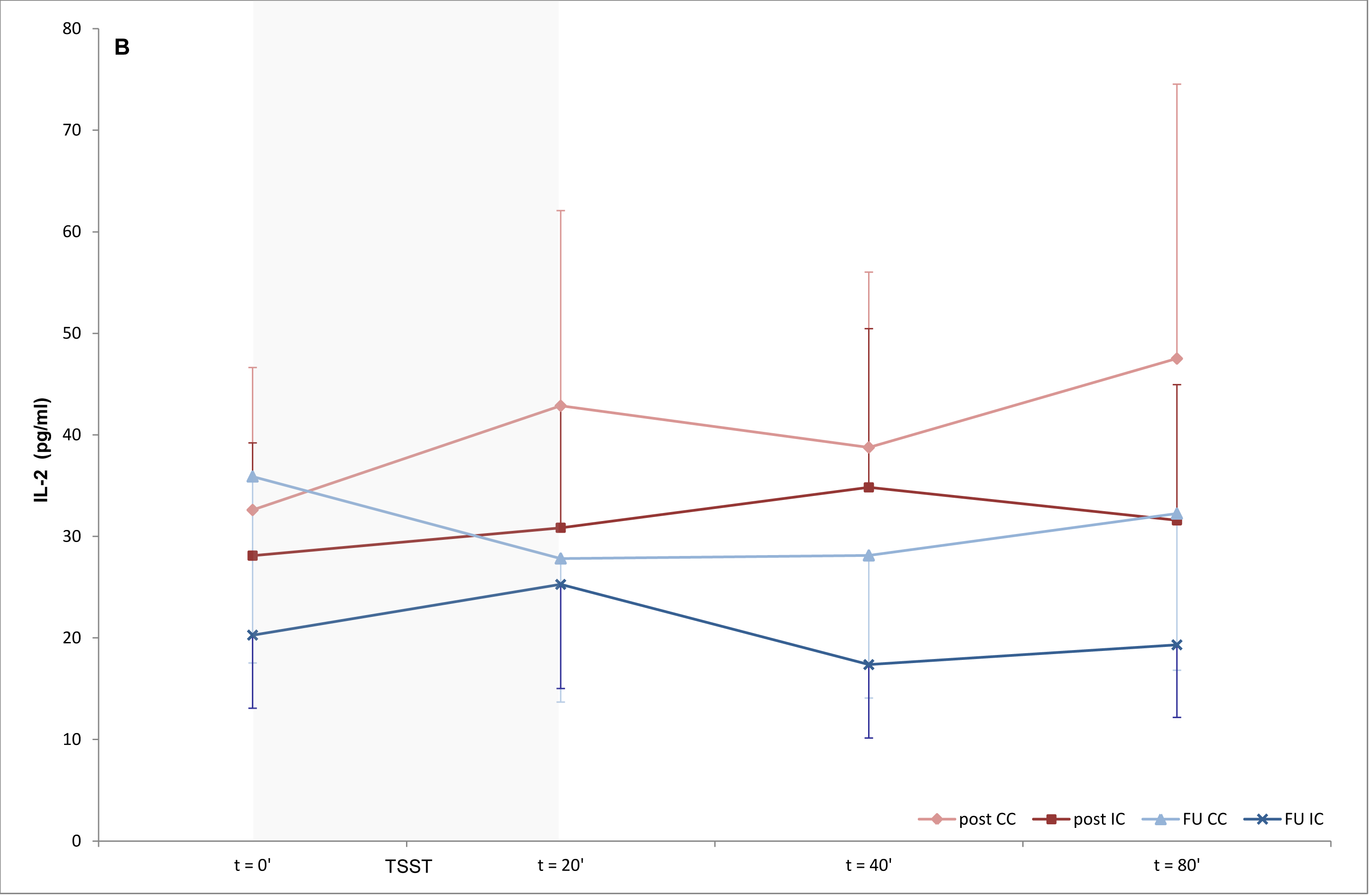

Supplement: Additional file 1 — is Figure S1 showing the mean response to stress of (A) IL-1β, (B) IL-2, (C) IL-4, (D) IL-5, (E) IL-6, (F) IL-7, (G) IL-8, (H) IL-10, (I) IFNγ, and (J) TNFα (in pg/ml ± standard error of the mean) at t = 0 minutes (baseline/pre TSST), t = 20 minutes, t = 40 minutes, and t = 80 minutes (post TSST) for patients in the intervention condition (IC) and control condition (CC) immediately after the intervention (post; red) and at follow-up (FU; blue). [file ar4390-S1.zip › Additional file 1/4354025511000446_add2.tiff]

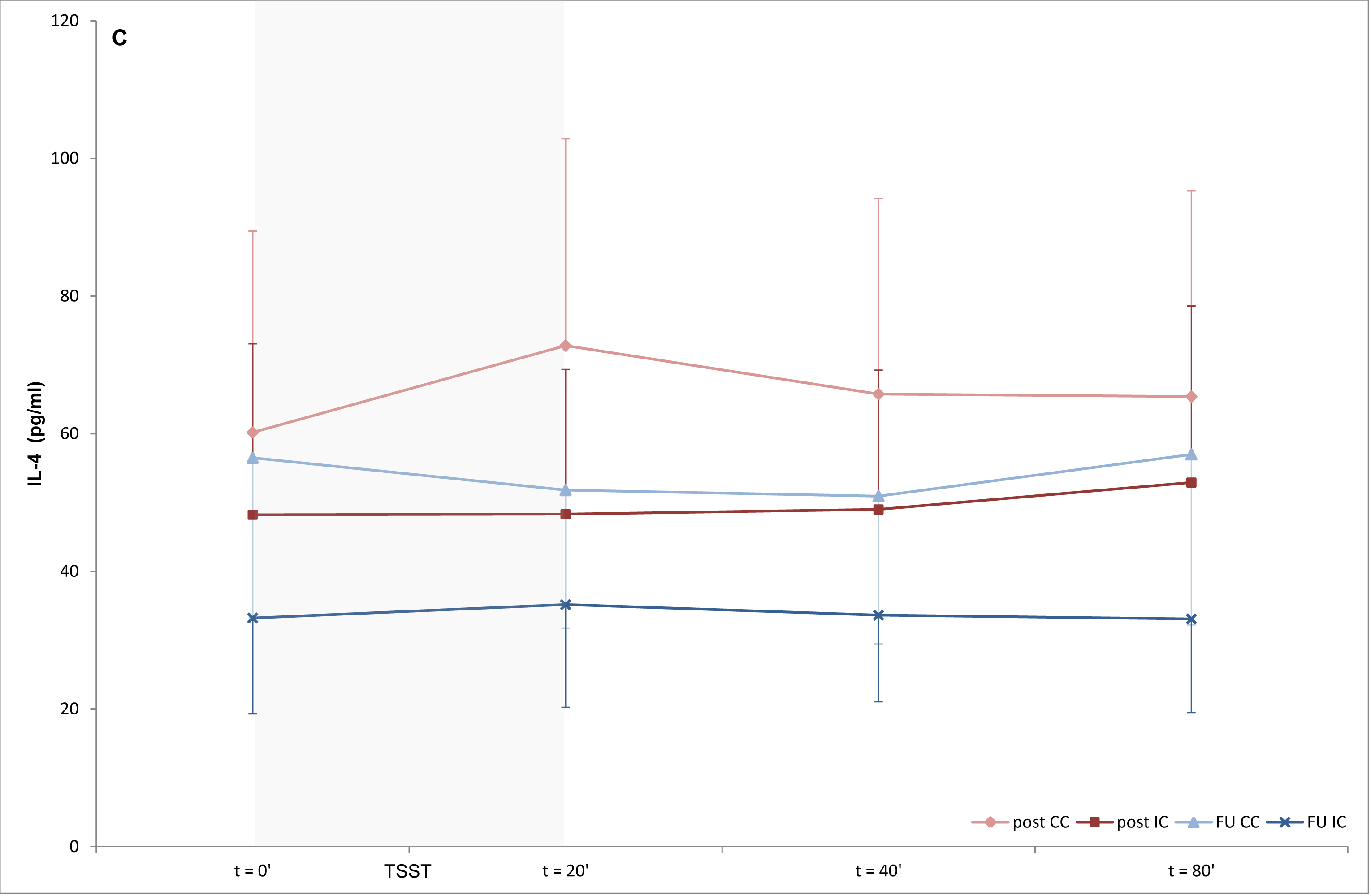

Supplement: Additional file 1 — is Figure S1 showing the mean response to stress of (A) IL-1β, (B) IL-2, (C) IL-4, (D) IL-5, (E) IL-6, (F) IL-7, (G) IL-8, (H) IL-10, (I) IFNγ, and (J) TNFα (in pg/ml ± standard error of the mean) at t = 0 minutes (baseline/pre TSST), t = 20 minutes, t = 40 minutes, and t = 80 minutes (post TSST) for patients in the intervention condition (IC) and control condition (CC) immediately after the intervention (post; red) and at follow-up (FU; blue). [file ar4390-S1.zip › Additional file 1/4354025511000446_add3.tiff]

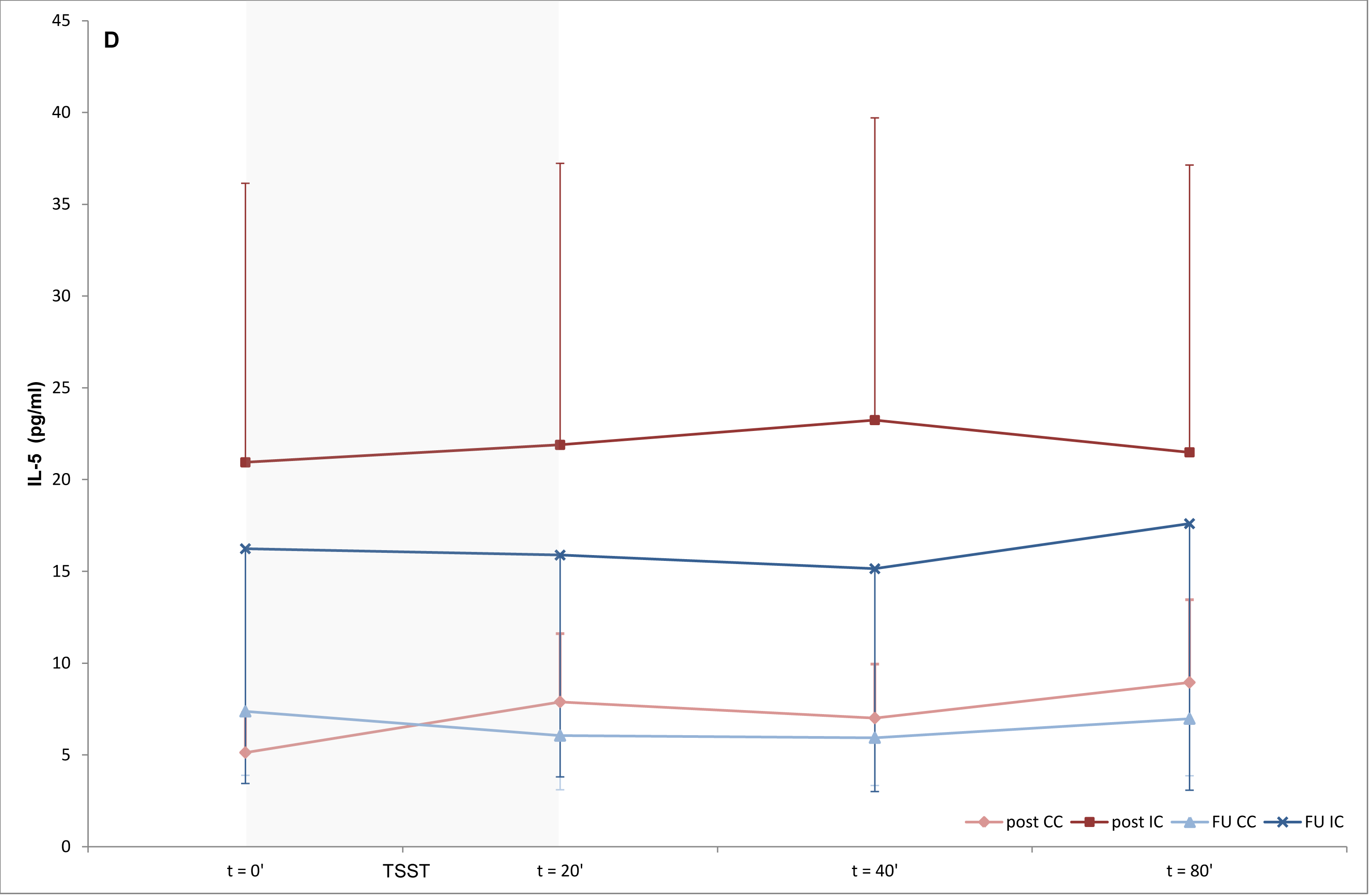

Supplement: Additional file 1 — is Figure S1 showing the mean response to stress of (A) IL-1β, (B) IL-2, (C) IL-4, (D) IL-5, (E) IL-6, (F) IL-7, (G) IL-8, (H) IL-10, (I) IFNγ, and (J) TNFα (in pg/ml ± standard error of the mean) at t = 0 minutes (baseline/pre TSST), t = 20 minutes, t = 40 minutes, and t = 80 minutes (post TSST) for patients in the intervention condition (IC) and control condition (CC) immediately after the intervention (post; red) and at follow-up (FU; blue). [file ar4390-S1.zip › Additional file 1/4354025511000446_add4.tiff]

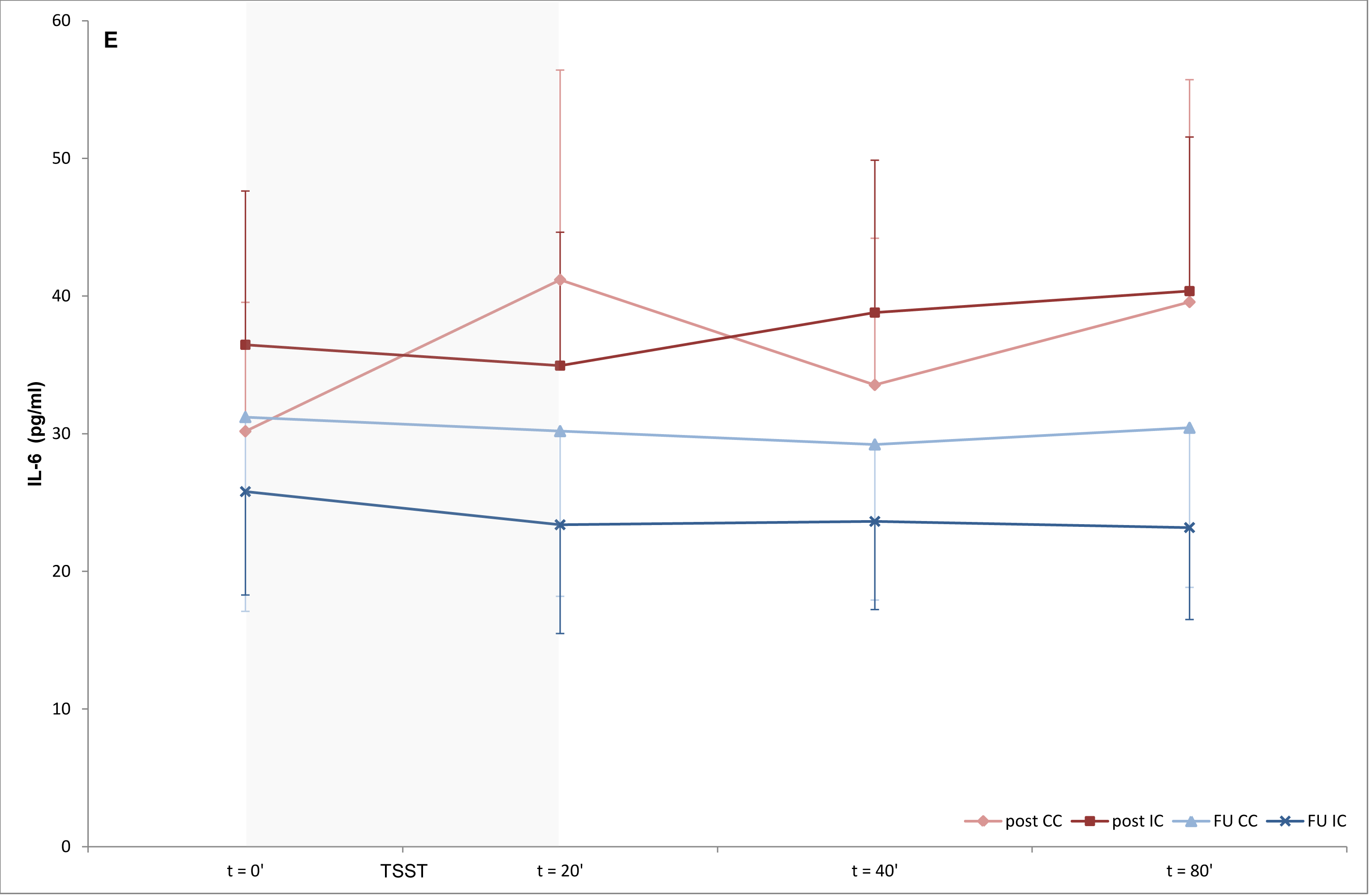

Supplement: Additional file 1 — is Figure S1 showing the mean response to stress of (A) IL-1β, (B) IL-2, (C) IL-4, (D) IL-5, (E) IL-6, (F) IL-7, (G) IL-8, (H) IL-10, (I) IFNγ, and (J) TNFα (in pg/ml ± standard error of the mean) at t = 0 minutes (baseline/pre TSST), t = 20 minutes, t = 40 minutes, and t = 80 minutes (post TSST) for patients in the intervention condition (IC) and control condition (CC) immediately after the intervention (post; red) and at follow-up (FU; blue). [file ar4390-S1.zip › Additional file 1/4354025511000446_add5.tiff]

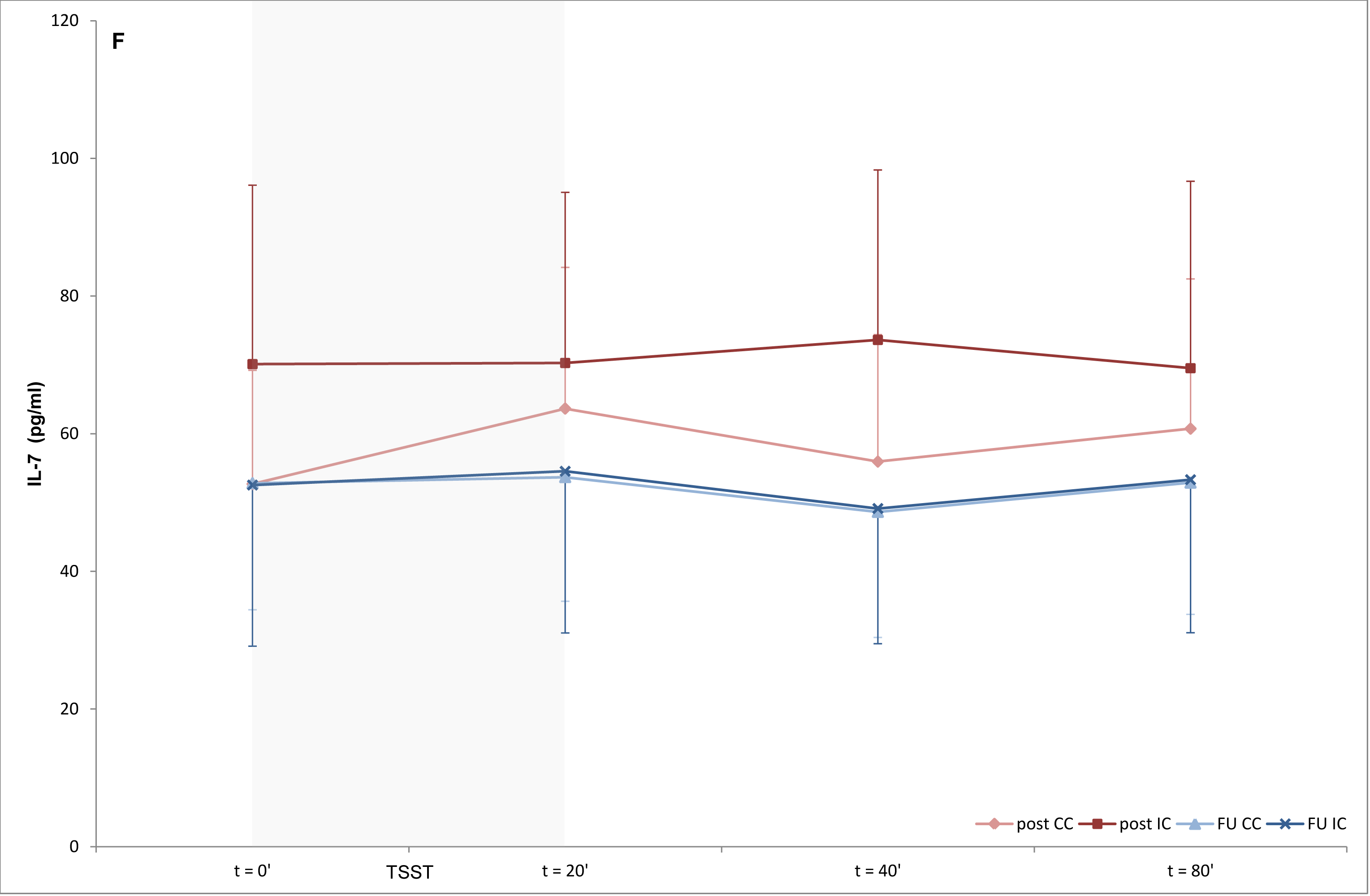

Supplement: Additional file 1 — is Figure S1 showing the mean response to stress of (A) IL-1β, (B) IL-2, (C) IL-4, (D) IL-5, (E) IL-6, (F) IL-7, (G) IL-8, (H) IL-10, (I) IFNγ, and (J) TNFα (in pg/ml ± standard error of the mean) at t = 0 minutes (baseline/pre TSST), t = 20 minutes, t = 40 minutes, and t = 80 minutes (post TSST) for patients in the intervention condition (IC) and control condition (CC) immediately after the intervention (post; red) and at follow-up (FU; blue). [file ar4390-S1.zip › Additional file 1/4354025511000446_add6.tiff]

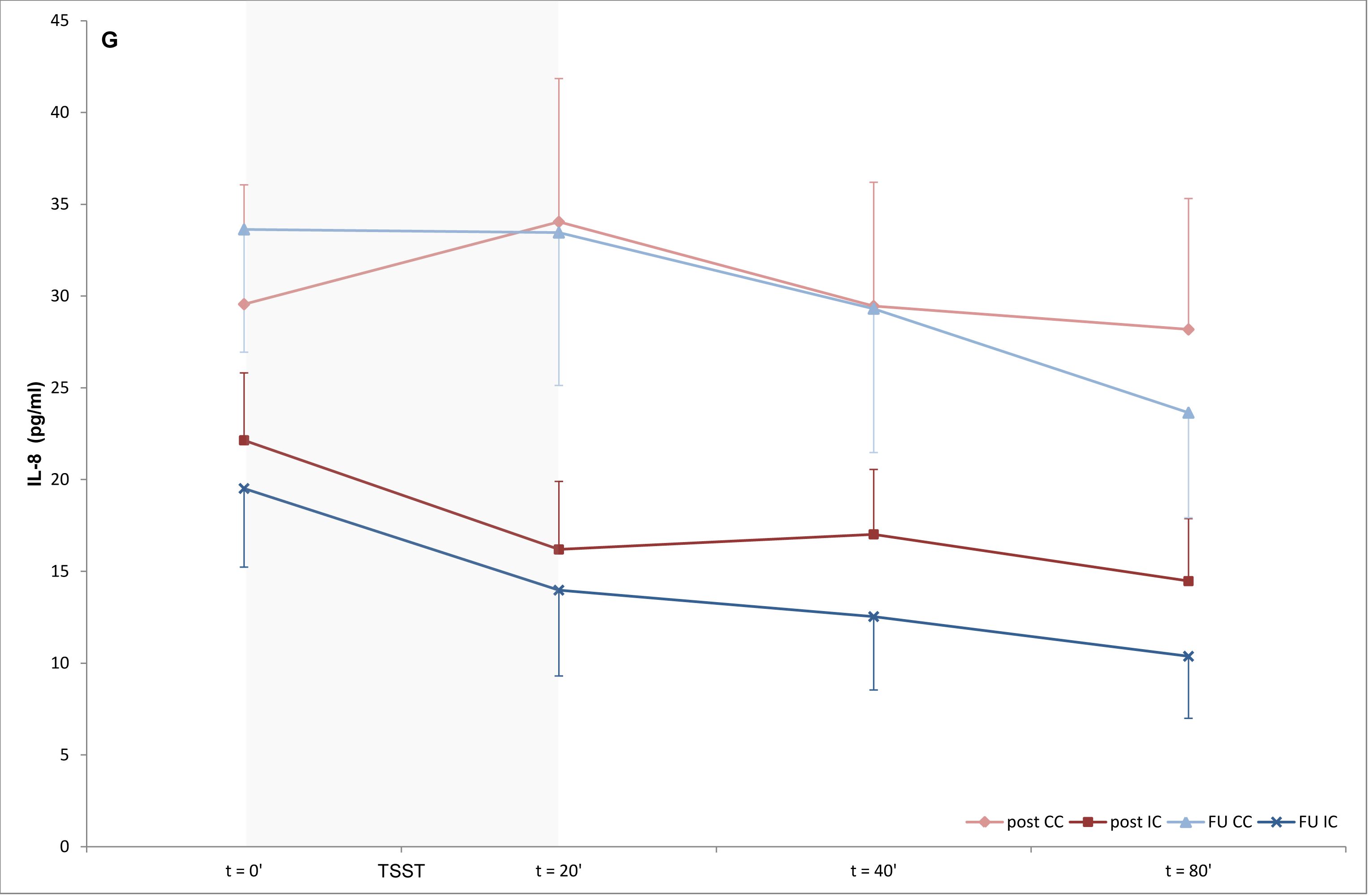

Supplement: Additional file 1 — is Figure S1 showing the mean response to stress of (A) IL-1β, (B) IL-2, (C) IL-4, (D) IL-5, (E) IL-6, (F) IL-7, (G) IL-8, (H) IL-10, (I) IFNγ, and (J) TNFα (in pg/ml ± standard error of the mean) at t = 0 minutes (baseline/pre TSST), t = 20 minutes, t = 40 minutes, and t = 80 minutes (post TSST) for patients in the intervention condition (IC) and control condition (CC) immediately after the intervention (post; red) and at follow-up (FU; blue). [file ar4390-S1.zip › Additional file 1/4354025511000446_add7.tiff]

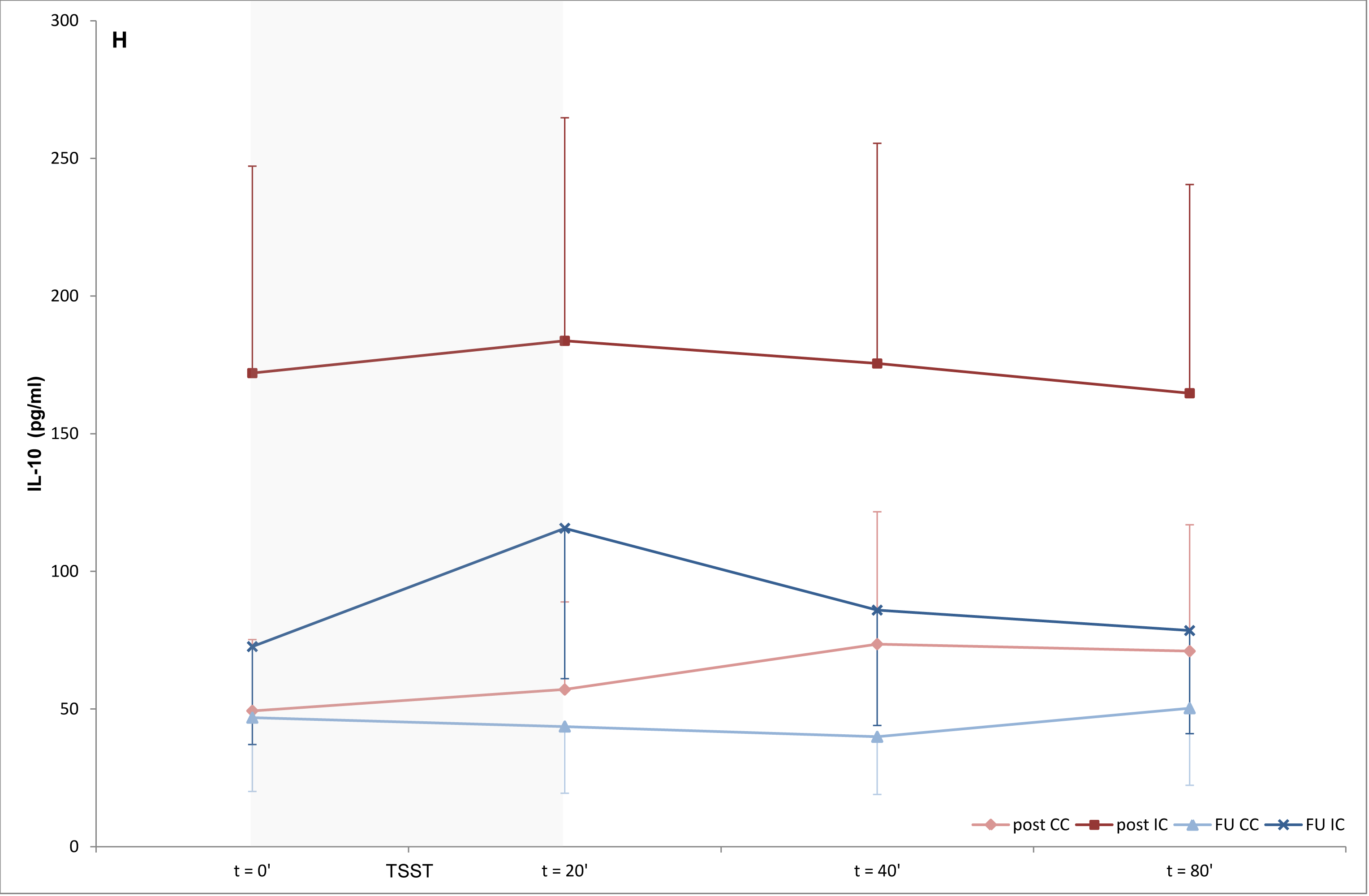

Supplement: Additional file 1 — is Figure S1 showing the mean response to stress of (A) IL-1β, (B) IL-2, (C) IL-4, (D) IL-5, (E) IL-6, (F) IL-7, (G) IL-8, (H) IL-10, (I) IFNγ, and (J) TNFα (in pg/ml ± standard error of the mean) at t = 0 minutes (baseline/pre TSST), t = 20 minutes, t = 40 minutes, and t = 80 minutes (post TSST) for patients in the intervention condition (IC) and control condition (CC) immediately after the intervention (post; red) and at follow-up (FU; blue). [file ar4390-S1.zip › Additional file 1/4354025511000446_add8.tiff]

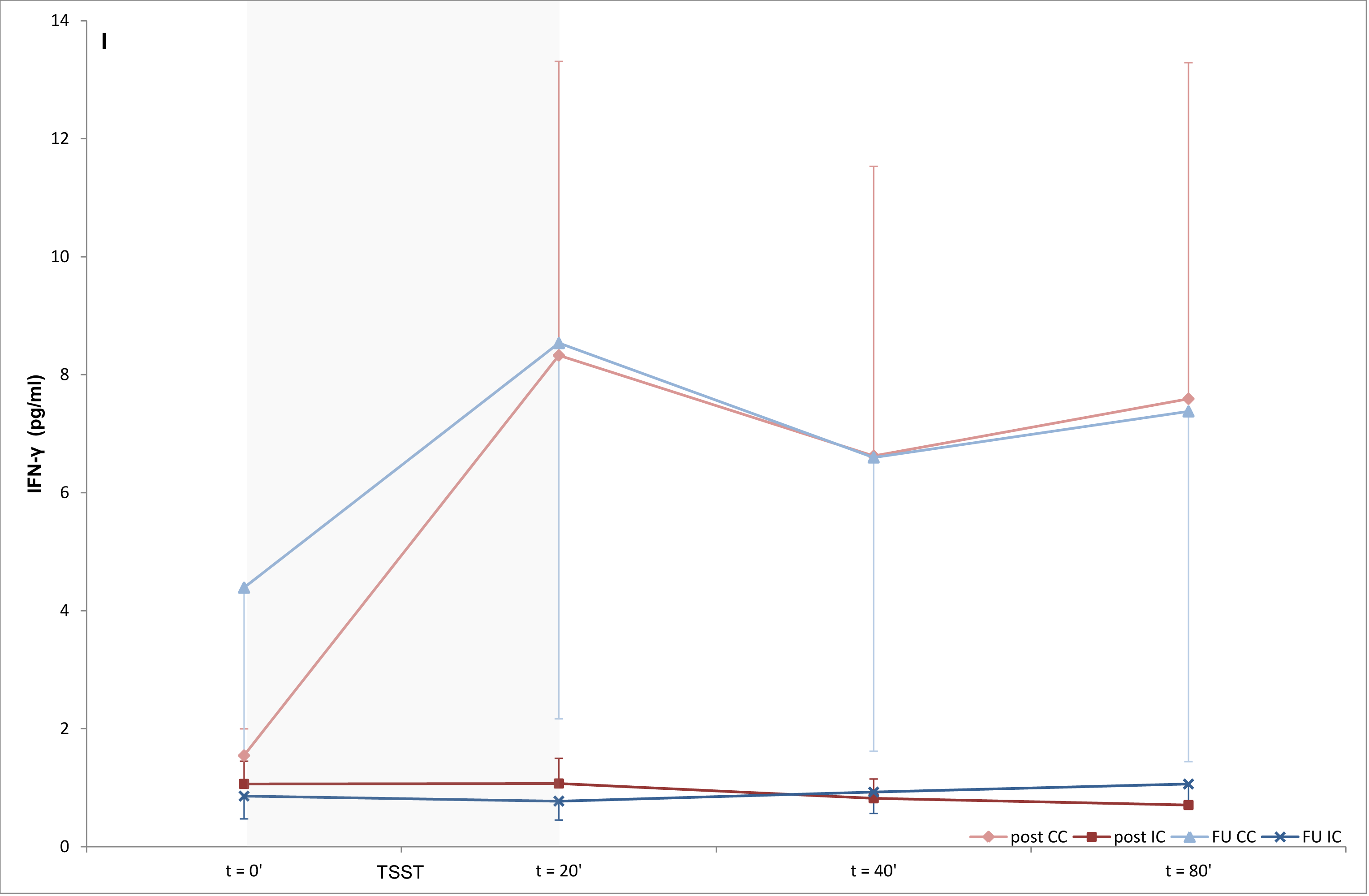

Supplement: Additional file 1 — is Figure S1 showing the mean response to stress of (A) IL-1β, (B) IL-2, (C) IL-4, (D) IL-5, (E) IL-6, (F) IL-7, (G) IL-8, (H) IL-10, (I) IFNγ, and (J) TNFα (in pg/ml ± standard error of the mean) at t = 0 minutes (baseline/pre TSST), t = 20 minutes, t = 40 minutes, and t = 80 minutes (post TSST) for patients in the intervention condition (IC) and control condition (CC) immediately after the intervention (post; red) and at follow-up (FU; blue). [file ar4390-S1.zip › Additional file 1/4354025511000446_add9.tiff]
